# Validation of an individualised quality of life measure in older day hospital patients

**DOI:** 10.1186/1477-7525-6-27

**Published:** 2008-04-18

**Authors:** Miles D Witham, Roberta L Fulton, Lucy Wilson, Carolyn A Leslie, Marion ET McMurdo

**Affiliations:** 1 Section of Ageing and Health, University of Dundee, Ninewells Hospital, Dundee DD1 9SY, UK

## Abstract

**Background:**

To test the ease of use, reliability, responsiveness and construct validity of the Patient Generated Index, an individualised quality of life score, in older people attending a Medicine for Older People Day Hospital.

**Methods:**

Prospective longitudinal study in patients attending a specialist Medicine for Older People Day Hospital in Scotland. The Patient Generated Index was administered at baseline, one week later, and at the end of Day Hospital attendance. Functional Limitations Profile, Hospital Anxiety and Depression Score, Barthel index and global subjective impressions of change were also collected and compared with baseline scores and change in Patient Generated Index scores. Reliability was assessed using intraclass correlation coefficients in subjects reporting no change in global quality of life; responsiveness was assessed using effect size and Guyatt coefficients in subjects reporting change in global quality of life. External validity was assessed via correlation with measures of physical function, comorbid disease and psychological state.

**Results:**

75 patients were enrolled, mean age 81 years. Mean completion time was 5.0 minutes at baseline. Reliability was moderate (intraclass correlation coefficient 0.72) but there were weak and inconsistent responses to change (effect sizes 0.02 to 0.15; Guyatt responsiveness coefficient 0.29). Patient Generated Index scores correlated with Functional Limitation Profile scores (r = 0.51, p < 0.001), baseline anxiety score (r = -0.25, *P *= 0.039) and baseline depression score (r = -0.37, *P *= 0.002) but displayed only weak, non-significant correlation with number of comorbid diseases (r = -0.22, *P *= 0.07), number of medications (r = -0.21, *P = *0.08) and Barthel score (r = 0.09, p = 0.45).

**Conclusion:**

The Patient Generated Index appears moderately reliable and easy to complete, but is poorly responsive to change, limiting its usefulness in clinical practice or research.

## Background

Quality of life is regarded as a key healthcare outcome by patients and by clinicians; both groups see improvement in quality of life as an important function of Medicine for Older People services such as Day Hospitals [1]. Most Day Hospital services do not routinely measure quality of life however; the emphasis remains on the measurement of function [2,3], and it is thus difficult to know whether such services are successful in improving quality of life.

Most 'quality of life' measures in current use focus either on health-related quality of life or health status – different concepts from overall quality of life [4]. Whilst it is generally thought that health is an important determinant of overall quality of life, and that health services are best placed to address only this aspect of quality of life, such a compartmentalised approach is at odds with the holistic ethos of health and social care that is central to the activities of Day Hospitals for older people.

Most quality of life tools in current use are further limited by the use of a set series of questions. Quality of life is a highly individual, subjective concept [5], thus a single set of questions may lack face validity for many patients – i.e. the questions may not cover areas of quality of life important to the individual patients. In an attempt to overcome this issue, and to broaden the measurement of quality of life beyond health issues, individualised quality of life tools have been developed [6-8]. The Patient Generated Index (PGI) is such a tool, deriving in part from the hypothesis that quality of life can be depicted as the fit between expectation and reality at a given time [5].

If Day Hospitals are to be effective in improving and promoting quality of life, it is important to try and measure quality of life. Individualised quality of life tools have been used successfully in a variety of clinical settings, including in older people [7-9] and may overcome some of the limitations described above. Before such tools can be used in clinical practice, they require to be tested in the population of interest. Such tools must be easy to use, reliable (stable over time in stable patients), responsive to change, and have validity (correlate with either gold standard measures or with other measures of function, environment and psychosocial status and experience) [10]. To be of use in clinical practice, tools should also collect data that are not collected by existing tools, and which have an impact on clinical activity.

Little previous work has been done using individualised quality of life tools in older people. We have previously shown that the PGI can be used in older people with heart failure, although the responsiveness of the tool was poor in this population. The SEIQoL has been studied in older hospitalised people [9], healthy older people [7] and in octogenarians in a longitudinal study [8]; the first group had some difficulties using the direct weighting device and took an impractically long time to use the tool. We were unable to find evidence that individualised quality of life tools have been assessed in a Medicine for the Elderly Day Hospital setting.

In this paper, we report the results of a study designed to test the acceptability, reliability, responsiveness and validity of an individualised quality of life tool – the Patient Generated Index – in a cohort of older patients attending a Medicine for the Elderly Day Hospital.

## Methods

### Patient selection and recruitment

Patients were recruited from the Medicine for Older People outpatient clinic at Royal Victoria Hospital, Dundee, UK, prior to their attendance at Royal Victoria Day Hospital. Patients are referred by primary care practitioners in the community or attend as follow-up after an in-patient stay in the hospital. Patients aged 65 years or over are referred with a wide range of presentations, including but not limited to falls, immobility, incontinence, breathlessness, weight loss and general debility. After assessment in the outpatient clinic, patients requiring multidisciplinary assessment and therapy attend the Day Hospital service once or twice a week. Attendance at Day Hospital usually lasts between four and six weeks.

Patients were eligible to be included in the study if they were due to attend Day Hospital. Patients with only a single planned Day Hospital attendance were excluded, as were those with a Folstein mini-mental state examination score of < 18/30, and those who were otherwise unable to give written informed consent. The study was approved by Tayside Local Research Ethics committee (application 05/S1401/107) and conformed to the principles of the Declaration of Helsinki.

### Study visits

Patients were given study information at their Medicine for the Elderly clinic appointment. Interviews were carried out during subsequent Day Hospital attendances to avoid the inconvenience of extra appointments or visits for the patient. Mini-mental state examination score [11] was recorded prior to obtaining written informed consent from all patients. The questionnaires administered were the Patient Generated Index [12] (higher score = better quality of life), the Functional Limitation Profile (FLP) [13] (a subjective measure of function; higher scores = worse function), and the Hospital Anxiety and Depression Score (HADS) [14] (higher scores = more anxious or depressed).

Questionnaires were administered at baseline (first visit to Day Hospital), one week later and at the final Day Hospital attendance. All questionnaires were administered by a trained nurse (RLF) with experience in caring for older people. Questionnaires were administered in the same order at each visit. At each time point, patients were asked to mark a visual analog scale to indicate their overall quality of life and at week one and discharge were also asked how their overall quality of life had changed since their last visit on a seven point Likert scale, ranging from 'much worse' through 'no change' to 'much better'. At the one week and final visits, patients answered the Patient Generated Index in two different ways. Firstly, they answered the questionnaire without reference to previous results. They were then presented with their selection of domains for quality of life from the baseline visit, which they rescored.

The Patient Generated Index has been described previously and has been tested in a variety of conditions including back pain, heart failure, colorectal cancer and postnatally [6,12,15-17]. Please see Additional File 1 for a description. Baseline data were obtained from the medical notes on age, sex, past medical history, patients' current medication as well as social circumstances. The Barthel Index, routinely collected by Day Hospital staff, was recorded on the first and last Day Hospital attendances.

### Sample size calculation

Pilot work suggested that the correlations between the FLP and PGI, and between the PGI and the Barthel Index, were approximately r = 0.4. To detect this degree of correlation at the 0.05 significance level with 90% power requires 50 patients (one-sided test). We assumed a dropout rate of 30% which would therefore have required 72 patients to be recruited. To detect a 25% improvement or deterioration in quality of life score during attendance at Day Hospital, given a baseline PGI score of 65.8 (SD 36) as found in our pilot work, a sample size of 52 would be required (1 sample students t-test, p < 0.05, 90% power). Allowing for a 30% dropout rate required 75 patients to be recruited to the study.

### Statistical analyses

Data were entered onto an Excel database and transferred to SPSS version 14 (SPSS, Chicago, USA) for statistical analysis. Reliability was assessed using those patients who reported 'no change' in quality of life on a Likert scale between baseline and one week. PGI scores at baseline and one week for this group were compared with intraclass correlation coefficients, using a one-way random effects model. Responsiveness was assessed using those patients who reported improvement or deterioration in overall quality of life on either the Likert scale or visual analog scale between week 1 and the final visit. Two methods of calculating responsiveness were used: a) effect size (test2-test1)/SD (test1) and b) Guyatt responsiveness coefficient, calculated as: minimally important difference/SD (change in scores of patients selecting 'no change' on Likert scale), where minimally important difference = mean of change scores in patients getting 'a little better' and 'a little worse' on the Likert score [18]. Construct validity was tested by correlating questionnaire scores with functional outcome scores and with FLP scores within each time point.

## Results

127 patients were screened for suitability. Of these, two were admitted to hospital on their first visit to Day Hospital, three had significant cognitive impairment, 10 had only a single planned Day Hospital attendance, 9 declined to return to Day Hospital and 19 declined to enrol in the study without giving a reason. 75 patients were recruited to the study between July 2006 and March 2007 thus achieving the recruitment target. Baseline details are shown in Table 1. Of note, patients at baseline had anxiety scores just above the mean compared to normative population data, but significantly higher depression scores than seen in the general population (equivalent to the 80^th^/85^th ^centile) [19].

**Table 1:** Baseline Patient Details

Detail	Value
Mean age (yrs) (*SD*)	80.8 (*5.9*)
Male sex	25/75 (33%)
Marital Status	Single 5/75 (7%)
	Married 30/75 (40%)
	Widowed 40/75 (53%)
Formal care input at home	25/75 (33%)
Median MMSE (*interquartile range*)	28 (*4*)
Living Status	Own home 50/75 (67%)
	Sheltered housing 25/75 (33%)
Mean number of comorbidities (*SD*)	7.7 (*2.4*)
Mean number of medications (*SD*)	6.6 (*3.3*)
Mean HADS anxiety score (*SD*)	6.9 (*4.8*)
Mean HADS depression score (*SD*)	6.9 (*4.1*)
Mean FLP physical score (*SD*)	316 (*68*)
Mean FLP psychosocial score (*SD*)	332 (*105*)
Mean FLP total score (*SD*)	1091 (*180*)
Median Barthel index (*interquartile range*)	18.5 (*3*)

HADS: Hospital anxiety and depression score. Worst = 21 for each domain

FLP: Functional limitation profile. Worst = 1652

MMSE: Mini mental state examination. Best = 30

Barthel: Best = 20

### Dropout rates

72/75 (96%) of subjects attended the one-week follow up appointment, and 63/75 (74%) attended the final follow-up appointment. Reasons for non-attendance were ill-health or hospitalisation (5 patients), early discharge from Day Hospital (6 patients) and one patient who declined further participation as they could not complete the questionnaires. Subjects attended Day Hospital weekly a median of 5 (interquartile range 2) times.

### Completion rates for the questionnaires

Completion rates were high for all questionnaires. 74/75 (99%) successfully completed the PGI at baseline, 72/72 (100%) completed successfully at week 1, and 63/63 (100%) completed the PGI successfully at the final visit.

### Time to completion for the PGI

Mean (SD) time to completion for the PGI was 5.0 (1.4) minutes at the baseline visit, 4.6 (1.6) minutes at the one week visit, and 4.2 (1.3) minutes at the final visit.

### Reliability

Reliability was scored by comparing baseline and 1 week scores in patients who had noted 'no change' in their overall quality of life at 1 week; a total of 40 patients. The intraclass correlation coefficient (ICC) was 0.72 (95% CI 0.54 to 0.84) for the PGI when new domains could be selected, and was 0.61 (0.37 to 0.77) for the PGI when the baseline domains were presented a week later.

### Responsiveness

Change scores for each category of change contained on the Likert score were calculated and compared. The categories 'worse' and 'much worse' were aggregated due to small numbers; similarly, 'much better' contained only one respondent and was aggregated with 'better'. Change in overall quality of life as marked on a visual analog scale was correlated with change in questionnaire scores between week 1 and the final week of attendance. Results are given in Tables 2 and 3.

**Table 2:** Observed Differences in Scores between Week 1 and Final Week compared with Likert scores

Change scores (*SD*)						
Domain	Worse/Much worse (*n *= 1)	A little worse (*n *= 3)	No change (*n *= 24)	A little better (*n *= 18)	Better/Much better (*n *= 17)	Spearmans rho

FLP physical	-28	-0.7 (1.1)	10.6 (53.2)	-4.5 (45.2)	-27.7 (49.1)	-0.267*
FLP psychological	7	33.0 (109.1)	12.4 (80.6)	-43.7 (75.4)	-15.7 (74.9)	-0.195
FLP total	-54	30.0 (105.5)	29.5 (124.2)	-32.4 (103.8)	-43.2 (95.5)	-0.262*
Barthel	-	0.67 (0.58)	0.24 (0.66)	0.29 (0.73)	0.67 (0.98)	0.098
PGI	0	5.0 (46.3)	-0.6 (30.6)	-9.4 (44.0)	13.1 (41.5)	0.141
PGI with original domains	0	-11.0 (39.7)	-7.8 (42.0)	-1.7 (35.6)	9.4 (37.6)	0.191

FLP: Functional Limitations Profile

PGI: Patient Generated Index

* *P *< 0.05

**Table 3:** Observed Differences in Scores between Week 1 and Final Week compared with visual analog scores

Domain	r (vs change in global QoL)	*p*
FLP physical	-0.065	0.61
FLP psychological	-0.070	0.59
FLP total	-0.048	0.71
Barthel	0.044	0.76
PGI	0.017	0.90
PGI with original domains	0.054	0.68

FLP: Functional Limitations Profile

PGI: Patient Generated Index

Effect sizes were calculated for patients who improved their overall quality of life between 1 week and the last visit at Day Hospital, and separately for those whose quality of life deteriorated. Results are shown in Table 4. Conventionally, an effect size of > 0.8 is regarded as large, 0.5 to 0.8 is moderate, and 0.2 to 0.5 is small. Responsiveness coefficients were also calculated for each tool; coefficients were 0.57 for the Barthel score, 0.26 for the FLP total, 0.29 for the PGI when new domains could be selected, and 0.004 for the PGI when baseline domains were presented at the final visit.

**Table 4:** Effect Sizes

Domain	Likert scale		Global Quality of life change	
	Improved (n = 35)	Deteriorated (n = 4)	Improved > 1 point (n = 23)	Deteriorated > 1 point (n = 14)

FLP physical	0.23	0.24	0.20	0.23
FLP psychological	0.28	0.27	0.16	0.21
FLP total	0.19	0.06	0.07	0.17
Barthel	0.24	0.58	0.23	0.22
PGI	0.04	0.15	0.04	0.02
PGI with original domains	0.11	0.23	0.02	0.27

FLP: Functional Limitations Profile

PGI: Patient Generated Index

### Construct validity

There is no gold standard with which to compare quality of life tools. However, common sense suggests that quality of life should worsen with worsening self-reported function and with worsening objectively assessed function. To test this, questionnaire scores were correlated with FLP and Barthel scores at each time point. The PGI showed moderate correlations with the FLP score (r = -0.44 to -0.51, *P *< 0.001) but weak correlations with the Barthel score (r = 0.09 at baseline, *P *= 0.45; r = 0.18 at final attendance, *P *= 0.2). The PGI was modestly correlated with the HADS anxiety score at baseline (r = -0.25, *P *= 0.039) and with the HADS depression score at baseline (r = -0.37, *P *= 0.002) but displayed only weak, non-significant correlation with number of comorbid diseases (r = -0.22, *P *= 0.07) or with the number of medications (r = -0.21, *P *= 0.08).

### Choice of domains

There was considerable variability in the domains chosen at both follow-up times with few subjects choosing the exactly the same domains at follow-up. After one week, 22/72 (31%) chose completely different domains of quality of life; at final follow-up, 15/63 (24%) chose completely different domains to those chosen at the baseline assessment. Domains chosen by participants are summarised in Table 5.

**Table 5:** Domains chosen by participants at each time point

	*Baseline (n = 75)*	*Visit 2 (n = 72)*	*Visit 3 (n = 63)*	*Total (%) (n = 210)*
Walking	36	38	23	97 (46%)
Independence	18	17	21	56 (27%)
Hobbies/interests	18	11	9	38 (18%)
Social life	15	11	11	37 (18%)
Sleep	16	13	6	35 (17%)
Shopping	12	12	11	35 (17%)
Climbing stairs	12	9	8	29 (14%)
Housework	9	10	7	26 (12%)
Getting outdoors	9	4	7	20 (10%)
Tiredness	6	9	3	18 (9%)
Pain/health	5	2	2	9 (4%)
Family	4	2	1	7 (3%)
Reading/writing	1	2	1	4 (2%)
Mood	3	0	1	4 (2%)
Looking after grandchildren	1	1	1	3 (1%)
Dizziness/headaches	1	0	2	3 (1%)
Unable to drive car	1	1	0	2 (1%)
Fear of falling	0	0	1	1 (0.5%)
War years	1	0	0	1 (0.5%)
Loneliness	1	0	0	1 (0.5%)
Rehousing (problem neighbour)	1	0	0	1 (0.5%)
Waterworks	0	1	0	1 (0.5%)
Sitting about	0	1	0	1 (0.5%)

## Discussion

This study has shown that the interviewer-administered Patient Generated Index (PGI) can be completed by the majority of older patients without significant cognitive impairment attending a Medicine for the Elderly Day Hospital. Completion rates were high, and the mean time to complete the tool was low. The reliability of the PGI was moderate, and interestingly the tool was less reliable when rescoring pre-chosen domains of quality of life. This suggests that re-presenting previously chosen domains of quality of life does not improve the psychometric properties of this tool, in contrast to findings with some other tools [20].

Responsiveness to change as measured by effect size was low for the PGI, although none of the tools used in this study displayed a large effect size. An effect size of > 0.8 is generally regarded as large, 0.5–0.8 is moderate, and 0.2 to 0.5 is small. Similarly, the responsiveness coefficients for the PGI suggested low responsiveness. Although correlation was evident between global change in quality of life as denoted by the Likert scale, correlations were weak and at times inconsistent – patients with worse overall quality of life scored similar PGI scores to those with improved global quality of life. There were no significant correlations between change in the PGI and change in the global visual analog score. This, in combination with the results from responsiveness to change analysis, suggests that the PGI cannot be relied upon as an index of change in quality of life in this patient population.

Correlation between the PGI and self-reported function (the FLP), anxiety and depression scores were moderate, suggesting that the PGI does have construct validity whilst collecting different information to the FLP. Correlation with the Barthel score was low and non-significant, suggesting that there was little overlap between the information collected by these tools, and that the PGI is collecting information that is not collected with standard Day Hospital assessment tools.

Strengths of our study include an adequately powered sample size and a representative group of attendees at Day Hospital including patients with mild to moderate cognitive impairment. We were able to test construct validity by comparing PGI scores with a variety of other measures of function and disease burden. Weaknesses of the study include the single centre nature of the study; results are not necessarily generalisable to other centres or other cultural settings.

Even though the PGI was originally designed to be a self-administered instrument, previous studies have found that interviewer administration of the PGI is necessary to ensure completion [12]. The PGI has previously found to have moderate reliability and evidence of construct validity has also been found [21-23]. The evidence regarding responsiveness is conflicting however; whilst some studies report reasonable responsiveness [15], others have noted a lack of responsiveness when using the PGI with older people [17]. The SEIQoL, a related individualised quality of life tool, has also accrued conflicting data regarding its responsiveness to change [24,25]. It is still possible however that such individualised quality of life tools are able to detect change, but that comparator measures used to assess this do not correlate with changes in quality of life. It has been suggested that over time, patients may change the way that they appraise their quality of life, which could mask changes in real quality of life unless the underlying process that the individual uses to assess their quality of life is also measured [26].

## Conclusion

This study has shown that the Patient Generated Index can be completed quickly and successfully when administered by interview to older people in Day Hospital. The tool showed moderate reliability, poor responsiveness to change, and variable external validity. Whilst the PGI may have some use as a baseline measure of quality of life, the lack of responsiveness makes it unsuitable for use as a measure of change in Day Hospital. The pursuit of individualised quality of life information remains a worthy goal however and future work should focus on ways of improving the responsiveness of the tool, whilst ensuring that it does not increase further in complexity.

## Competing interests

The authors declare that they have no competing interests.

## Authors' contributions

LW, CAL, METM and MDW produced pilot data and designed the study. RLF collected the data and helped analyse the data. MDW analysed the data and wrote the paper. All authors contributed to the writing and revising of the paper. All authors read and approved the final manuscript

## Supplementary Material

Additional file 1 PGI and description. A copy of the PGI as used in the study, together with a description of its use. Click here for file
