# Supplementary material for: Validation of an individualised quality of life measure in older day hospital patients
Source: Health Qual Life Outcomes. 2008 Apr 18;6:27. doi: 10.1186/1477-7525-6-27 (PMC2373778; doi:10.1186/1477-7525-6-27)
Supplement: Additional file 1 — PGI and description. A copy of the PGI as used in the study, together with a description of its use. [file 1477-7525-6-27-S1.doc]

**Appendix 1. Description and proforma for the Patient Generated Index**

The tool is divided into three sections. In the first section, patients select up to five areas of importance in their lives. Two extra categories are also provided; areas affected by other health problems, and all other non-health areas of life. Patients are then asked to score their quality of life in each of these seven domains, with 10 representing quality of life exactly as they wanted to be and 0 the worst they could imagine. In section three subjects are given the opportunity to spend fourteen imaginary points to improve the seven domains. They were asked to spend more points improving areas that were important to them and to spend less or nothing on areas of less importance. Each score in section two is multiplied by points awarded in section three, divided by 14, and the seven domains are summed. Low scores thus represent low quality of life and the maximum score is 140.

# Patient Generated Index

## STEP 1: Identifying areas

We would like you to think of the most important areas of your life. Please write up to FIVE areas in the boxes below.

Here are some areas other patients have mentioned that might help you to think how your life has been affected:

Social life, work, housework, hobbies, interests, mood, loss of independence, can’t go shopping, walking, climbing stairs, sitting, sleep, tiredness

AREAS AFFECTED BY OTHER HEALTH PROBLEMS

ALL OTHER NON-HEALTH AREAS OF LIFE

## STEP 2: Scoring each area

In this part we would like you to score the areas you mentioned in step 1. This score should show how badly affected you were over the past MONTH. Please score each area out of 10 using this scale:

10 Exactly as you would like to be

9 Close to how you would like to be

8 Very good but not how you would like to be

7 Good, but not how you would like to be

6 Between good and fair

5 Fair

4 Between poor and fair

3 Poor but not the worst you could imagine

2 Very poor but not the worst you could imagine

1 Close to the worst you could imagine

0 The worst you could imagine

Please use the last two boxes to score all areas affected by other health problems and all other non-health areas of life

## STEP 3: Spending points

We want you to imagine that any or all the areas of your life could be improved. You have 14 imaginary points to spend to show which areas you would most like to see improve. Spend more points on areas you would most like to see improve and less on areas that are not so important.

You don’t have to spend points in every area. You can’t spend more than 14 points in total.

Remember total must add up to 14
